# Prosthetist Knowledge and 3D Printing

**DOI:** 10.33137/cpoj.v7i2.42175

**Published:** 2024-12-14

**Authors:** M Ratto, D Southwick

**Affiliations:** 1 Faculty of Information, University of Toronto, Toronto, Canada.; 2 Autodesk inc., San Francisco, California, USA.

**Keywords:** Prosthetics, Orthotics, Fabrication, Additive Manufacturing, 3D Printing, Design, Automation, Knowledge, Prosthetist, Orthotist

## Abstract

In this paper we briefly explored the history of 3D printing in prosthetics. We provided details of our own work developing 3D printing design tools from 2014–2020 noting how claims around prosthetist experience and knowledge have been supported and/or questioned in the development of new device production techniques. We ended by arguing for deeper attention to prosthetist knowledge and experience in the design of the growing 3D printing ecosystem, seeing this focus as necessary and important to preserve and support clinical prosthetist in the production of well-fitting and appropriate devices for patients.

## INTRODUCTION

In this paper we briefly explored 3D printing and prosthetics, understanding 3D printing as connected to but distinct from the long history of CAD/CAM technologies in P&O. Work on digital production of prosthetics began in the 1960's but despite the development of multiple systems over the last 70 years, most prosthetic devices are still produced through craft processes. If asked, most prosthetists will note the existence of automated systems, but highlight their inadequacies in coming to terms with the complex topologies and textures of a humans and their cost and difficulty of use.

Recent technological developments, including lower cost 3D scanners with higher accuracies and easier to use 3D printers capable of printing in higher tensile strength materials offer the possibility of overcoming these stated challenges. However, developing digital prosthetic toolchains that properly acknowledge and instantiate forms of knowledge that constitutes prosthetist expertise is a long-standing challenge in the development of novel CAD/CAM prosthetic systems.

## 3D PRINTING AND PROSTHETICS

In 1990, a research group at Northwestern University, in conjunction with Baxter Healthcare, made a single trans-tibial (TT) socket using a form of 3D printing known as stereolithography (SLA).^1^ Shortly thereafter, the University of Texas at Austin and the University of Health Science Centre at San Antonio began experimenting with Selective Laser Sintering (SLS), which led to an amputee briefly wearing a 3D printed socket in a controlled clinical setting in 1992.^2^ While quite limited in scope, these early studies in the application of 3D printing within the P&O profession had a large impact in the profession. In the conclusion of the 1992 report written by the Department of Veteran Affairs (VA) on the Automated Fabrication of Mobility Aids (AFMA), which was discussed in detail in the previous chapters, six areas of research concentration are suggested. Of these six, one specifically called for further research into methods for “automating the prosthesis manufacturing process”, and the eventual direct prosthesis manufacturing from CAD files through “rapid prototyping CAM technologies”.^3^ The reasoning behind this move was that “rapid prototyping CAM technologies”, or 3D printing, seemingly allowed for the manufacturing of prosthetic devices without the interruptive craft methods used in the traditional methods during the fabrication of the device.

A new kind of study into the application of 3D printing in P&O work emerged in the late 2000s, which differed from earlier studies in that they were a direct response by the P&O community to developments in technology and to new non-expert actors in the field. The introduction of the MakerBot Cupcake CNC and the Thing-O-Matic, in 2009 and 2010 respectively, marked an important turning point in the development of 3D printing technology from both a cost and usability perspective.^4^ Under such slogans as “If you can think it, you can make it” Makerbot began to actively promote the concept that “desktop manufacturing” was leading to a future of de-centralized production. This led to various hardware and software developments that not only further reduced the costs of 3D printing, but also made the technology far more accessible to users without backgrounds in engineering. These cheaper and more accessible 3D printers created by companies like Makerbot effectively addressed the major issue of cost associated with the technology. Yet, in doing so, a new type of problem was introduced in the form of non-expert actors developing digital tools and prosthetics.

In the late-2000s various organizations with little to no connection to the P&O profession began to use 3D printers to provide cheap and easily accessible prosthetics for both the developed and developing world by allowing users to fabricate their own devices.^5^ These desktop printable prosthetic devices were mainly upper extremity prostheses such as body-powered “hands” such as the Raptor Hand developed by Project E-nable.^6^ The P&O profession responded to these devices by studying them in various contexts. While acknowledging that these devices were useful for training patients to wear devices, on a functional level they were severely lacking. The durability of these prosthetics devices also proved problematic, with joints and areas of pressure frequently breaking.^7,8^ Finally, and most importantly from the perspective of the P&O profession, was the fit of these devices. Most of these devices used simple measurements to scale 3D printed components. The Raptor Hand, for example, is a device that uses three measurements on the wrist and the palm to determine the size of the prosthetic device.

In their critiques of this approach the P&O community note that this not only limits the “kinds” of amputations these devices can be used for, but it also fails to properly transfer the biomechanical forces that act on the limb.^7,8^ While the overall assessment of DIY prosthetics within the P&O community is fairly negative, those who have examined these devices often acknowledge the potential of 3D printing technology for the profession. Thus, the challenge going forward is to develop a system that better understands the various nuances of the P&O profession, while also leveraging the potential benefits of these technologies. This, in of itself, is a substantial undertaking. What has made this task even harder is the popular perception of 3D printing and prosthetics as a “solved problem” that formed due to the extensive media coverage of DIY prosthetics organizations.^9^ Unfortunately, this popular perception often involves a reduced role for prosthetist expertise in a 3D printed future in no small part due to a lack of understanding of the clinical and material nature of prosthetics themselves.

## 3D PRINTABILITY

**Figure 1:**
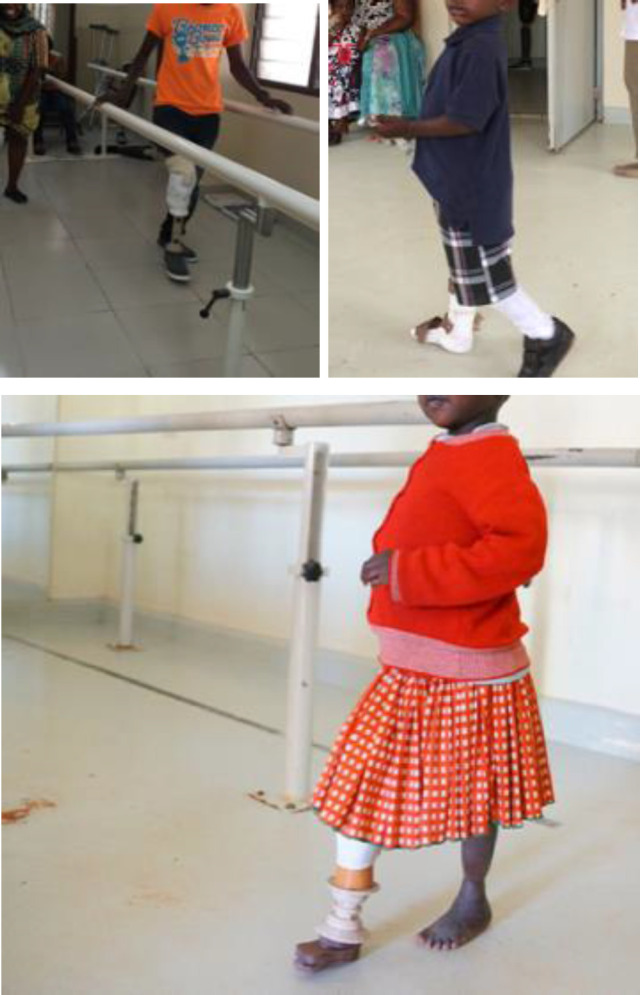
Images of patients from Uganda and Tanzania, 2018.

Since 2014, the authors of this paper have been part of a project called ‘3D PrintAbility’, developing 3D design and printing tools for use in rehabilitation clinics in low to middle income countries in Sub-Saharan Africa and Southeast Asia. Supported initially by a charitable organization and later through award-based funders including Grand Challenges Canada, Autodesk Foundation, and a Google Impact award, the team has created and deployed multiple versions of a software and hardware toolchain to produce pediatric lower-limb prosthetic and orthotic devices. More than 20 prosthetists and prosthetic technicians have participated in design exercises and been trained in 3D scanning, design, and printing technologies. Clinics that have partnered in this work include Comprehensive Rehabilitation Services for People with Disability in Uganda (CorSU) hospital, Comprehensive Community Based Rehabilitation Tanzania (CCBRT) and Tanzania Training Centre for Orthopaedic Technologists (TATCOT), and the Cambodian School of Prosthetics and Orthotics (CSPO) (**Figure 1**).

Nia Technologies (http://www.niatech.org), a non-profit organization located in Toronto, Canada currently provides support and continues to improve software and hardware solutions for P&O. Three clinical trials were carried out during the project, an initial trial at CorSU in Uganda, followed by a multi-site trial that included each of the partner sites described above. A third and smaller trial was also conducted in Canada at St. John's Rehabilitation Clinic/Sunnybrook Hospital in Toronto. A more detailed description and accounting of this work can be found in our previous publications.^10–13^ This paper will focus only on the 3D design software developed during the project.

The goal of 3D PrintAbility was to extend the traditional fabrication and fitting processes for lower-limb prosthetic sockets with 3D design software and hardware. As is well-known to the readers of this journal, the traditional socket fabrication and fitting process involves a three-step process. In the first step a “negative model” of the residual limb in created using plaster wraps. Next, a positive model is created by pouring plaster material into the negative model, allowing it to harden, and removing the wraps. This positive model is then “rectified”, a process where prosthetists add and subtract material to create a shape that, when used to produce a socket, will properly distribute weight and pressure across the residual limb. In the final step, a socket is fabricated over the positive model, using thermoplastics or lamination processes. Finally, the socket is fitted to the patient after some light post-production, such as sanding rough edges. During the actual fitting process, minor modifications can be made to the socket based on feedback from the patient. If, however, major modifications are required, the entire process must begin again. Similarly, when a patient requires a new socket, the entire process begins again. 3D PrintAbility was developed to reduce the time to completion of a prosthetic socket and by doing so to increase the number of patients that could be treated by rehabilitation clinics in low resource areas. This goal has many similarities to the reasons for the original development of digital fabrication technologies for P&O which included a strong desire to better capture the skills and expertise used in the development of prosthetic sockets.^14–17^

## 3D PRINTABILITY AND “SOCKET MIXER”

**Figure 2:**
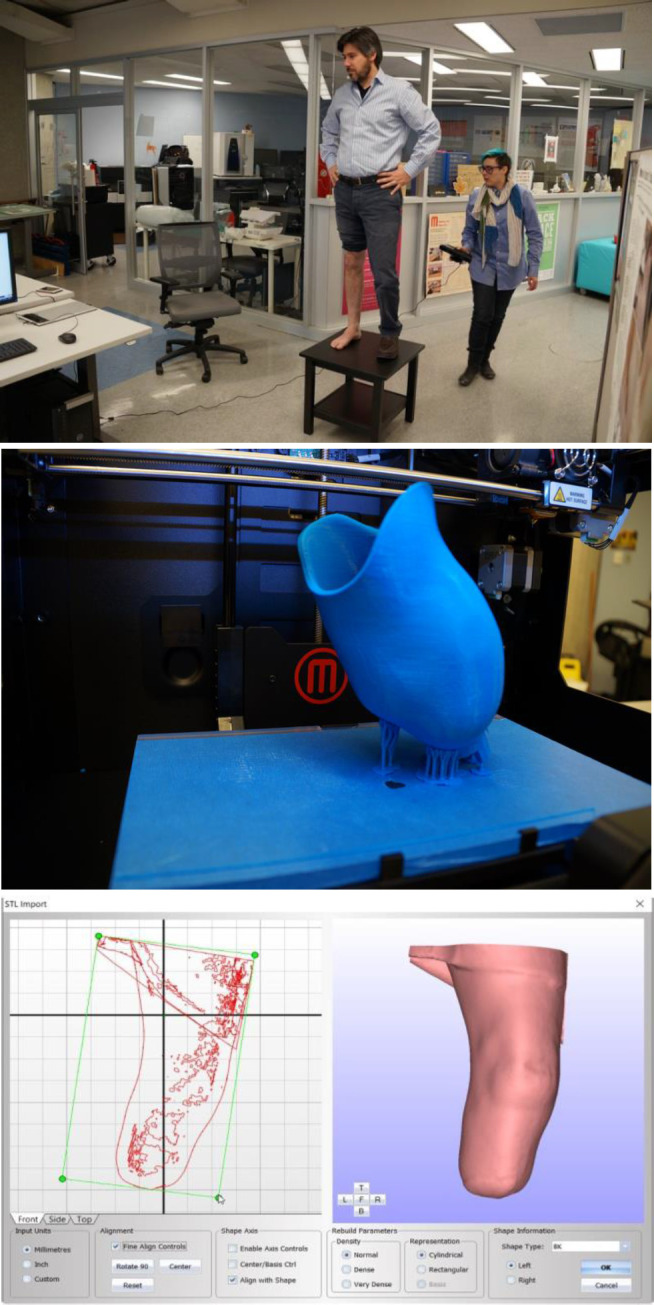
Author being scanned and printed socket (Top and Middle); CanFit software (Bottom).

In 2014, after initial experiments in 3D scanning, design, and printing (**Figure 2**), interviews with prosthetists and prosthetic users in Canada and Uganda, the 3D PrintAbility team began work on 3D design software made to supplement an existing CAD/CAM solution called CanFit, made by Vorum inc.^18^ While CanFit could be used to modify 3D scans and produce subtractive CNC milling patterns to produce positive models, at that time no prosthetist-specific software programs existed that could produce 3D printable prosthetic sockets.

**Figure 3:**
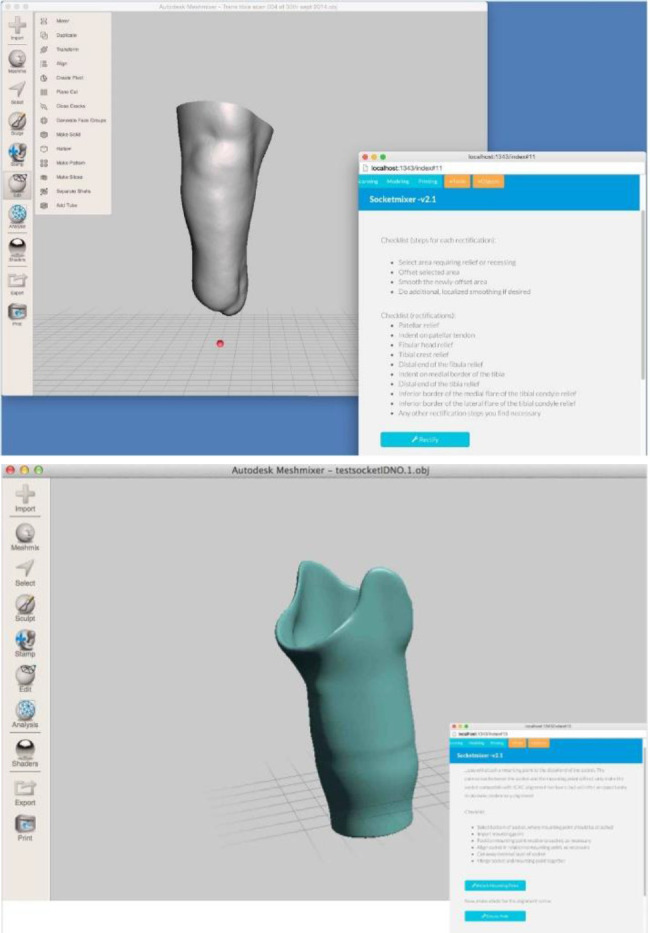
SocketMixer, Nia technologies, 2014.

The first version of this software we called ‘SocketMixer’. It worked as an add-on to the popular Autodesk MeshMixer free 3D software package^19^ and created an additional menu panel with custom commands focused on socket production. (**Figure 3**)

Our goal with this software was to simplify the complex chain of commands that could be used by a skilled engineer to convert a digital scan to a socket. However, this solution did not involve an automatic conversion from digital data to 3D printable socket, but instead allowed prosthetists to make key decisions about the resulting socket, including supporting the manipulation of the model to expand or reduce specific areas, control the thickness of the socket and the shape of the brim, and the location and shape of the mounting point for attaching the socket to the rest of the prosthetic device. Our goal in creating this simple software was to reduce complexity of use while still allowing the prosthetists own style and knowledge to be utilized.

## NIAFIT AND PROSTHETIST KNOWLEDGE

Our initial work with CanFit and on SocketMixer demonstrated the potential of 3D printing for 3D printing prosthetic sockets. It also revealed the difficulty for prosthetists to use multiple software programs to produce a socket. In our initial solution, prosthetists needed to start with from one program for scanning, move the resulting file to a different program for scan cleanup, still another for modeling, and yet another for the actual preparation of a printable file. This complexity created many opportunities for error and required prosthetists to learn major new skills to produce good sockets. To solve this issue, we decided to produce a more integrated software solution in which a single program provided all capabilities. Extended development, many tests and design research with prosthetists, and multiple years of testing resulting in NiaFit, a scanning, modeling, and printing suite.

**Figure 4:**
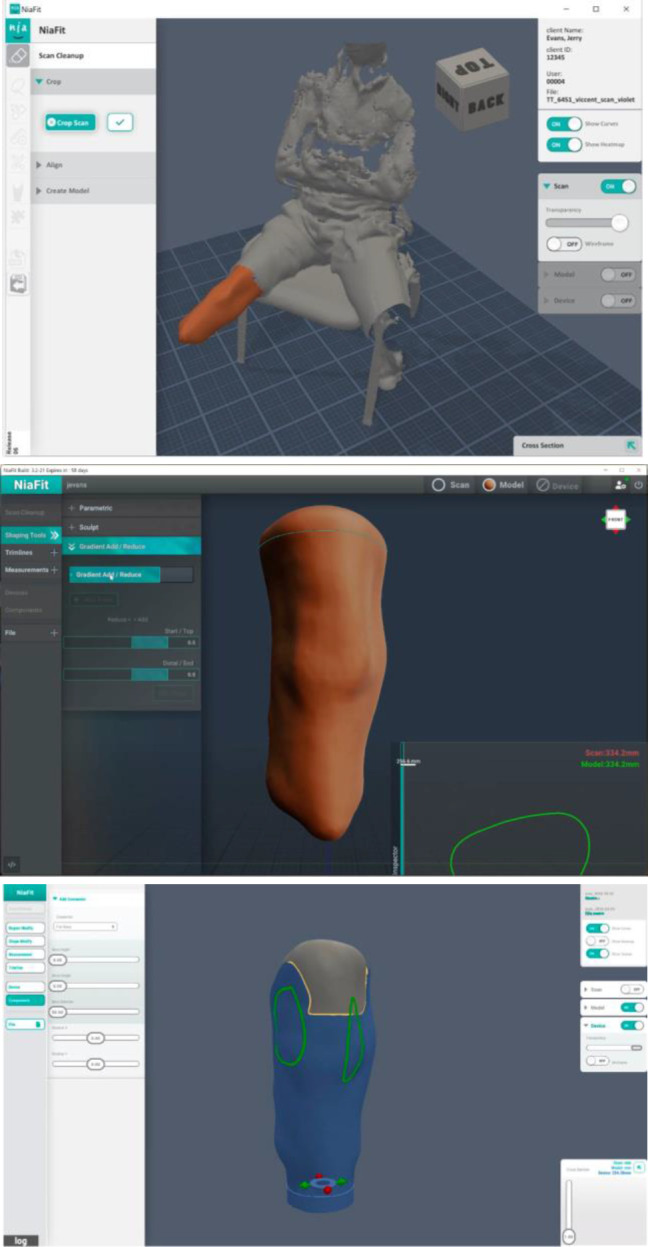
Three main screens and functions in NiaFit, 2018

As is shown in **Figure 4**, the user flow was separated into three main screens. First, a scan taken using an iPad + Structure scanner (Occipital inc.) was imported into NiaFit. The initial interface focused on tools needed for reducing the complexity of the scan and preparing the file for modeling activity. Clicking with a mouse on one area of the scan selects it (highlighted in orange in **Figure 4**) and the forward and back arrow keys extend the scan to contiguous areas. When done, all unselected parts of the scan are removed, and the resulting 3D shape is realigned within the view window. At this point, modeling tools become available, that allow focused and overall smoothing, as well as the building up or reducing of regions on the model. Other functions are also available, such as the creation of trimlines and the taking of measurements. When ready, the user moves to the final stage of the process. Here, tools are available that reproduce the process of ‘draping’ thermoplastics to produce a socket.

In developing NiaFit, three concerns were paramount. First, as noted above, we wanted to create a single application to reduce the complexity of the digital socket development process. Second, we chose to structure the digital user experience to reproduce as much as possible the traditional process of prosthetic socket production. By designing the experience in this way, the goal was to reduce the retraining necessary for prosthetists to transition to a digital workflow and to conserve their current knowledge and ways of working. Third, we wanted to help prosthetists extend and communicate their knowledge. A key insight by early developers of CAD/CAM systems in prosthetists was that such systems could make prosthetist skill and expertise more visible and reproduceable than the destructive craft-based processes then – and still – in use.^16^ In NiaFit, all prosthetist operations are preserved as separate digital ‘moves’, allowing for users to move back and forth within the prosthetic socket production process. Importantly, such ‘moves’ allow both the sequential ‘undo’ and ‘redo’ that is typical of digital workflows, but also the ability to remove or rework operations out of sequence, with the topology of the positive model or digital socket adjusting automatically. Such processes allow novel forms of ‘branching’ in prosthetic design and also foster and support the future development of more collaborative forms of prosthetic design and production. We developed this functionality based on our ongoing collaboration with working prosthetists who sought these new capabilities. Only recently have more mainstream CAD/CAM design tools begun to incorporate similar branching^20^ and collaboration^21^ features.

## CALL TO ACTION

Despite over 70 years of work on CAD/CAM and over 30 years since the first 3D printed prosthetic socket, these technologies remain undeveloped and of limited current use in mainstream P&O. New technical developments, including inexpensive scanners and printers capable of higher resolutions and stronger materials, are now available, and these may increase adoption. However, we strongly believe that design software needs to be developed in ways that foster, support, and extend prosthetist knowledge and expertise. This is best done with the participation of prosthetists' themselves who should drive the creation of new systems and platforms to best serve their patients.

## DECLARATION OF CONFLICTING INTERESTS

The lead author was the chief scientist of Nia Technologies, the non-profit formed to continue development of 3D design software from 2015-2020 and received compensation in this role. He served as the PI on all grants associated with its development.

## AUTHOR CONTRIBUTION

Both authors contributed equally to the research and the writing of this manuscript.

## SOURCES OF SUPPORT

This work was supported under the 3D PrintAbility Project funded by the Inclusive Design Institute; 2011 Canada Foundation for Innovation; 2014 Canada Grand Challenges Stars in Global Health; 2016 Canada Grand Challenges Transition to Scale; 2016 Google Impact Award; 2016 MITACS Award.
